# Systems Pharmacology-based strategy to screen new adjuvant for hepatitis B vaccine from Traditional Chinese Medicine *Ophiocordyceps sinensis*

**DOI:** 10.1038/srep44788

**Published:** 2017-03-20

**Authors:** Jingbo Wang, Rui Liu, Baoxiu Liu, Yan Yang, Jun Xie, Naishuo Zhu

**Affiliations:** 1 Laboratory of Molecular Immunology, State Key Laboratory of Genetic Engineering, Institute of Biomedical Science, School of Life Sciences, Fudan University, Shanghai, 200438, China

## Abstract

Adjuvants are common component for many vaccines but there are still few licensed for human use due to low efficiency or side effects. The present work adopted Systems Pharmacology analysis as a new strategy to screen adjuvants from traditional Chinese medicine. *Ophiocordyceps sinensis* has been used for many years in China and other Asian countries with many biological properties, but the pharmacological mechanism has not been fully elucidated. First in this study, 190 putative targets for 17 active compounds in *Ophiocordyceps sinensis* were retrieved and a systems pharmacology-based approach was applied to provide new insights into the pharmacological actions of the drug. Pathway enrichment analysis found that the targets participated in several immunological processes. Based on this, we selected cordycepin as a target compound to serve as an adjuvant of the hepatitis B vaccine because the existing vaccine often fails to induce an effective immune response in many subjects. Animal and cellular experiments finally validated that the new vaccine simultaneously improves the humoral and cellular immunity of BALB/c mice without side effects. All this results demonstrate that cordycepin could work as adjuvant to hepatitis b vaccine and systems-pharmacology analysis could be used as a new method to select adjuvants.

Adjuvants could elicit immune responses through different signaling pathways thus improve vaccine formulations for better protection^1^, however, there are still very few adjuvants have been licensed for human because of the side effects or other problems^2^,^3^,^4^. Even the widely used alum adjuvant could cause local reaction and IgE responses during vaccination. Therefore, there is a requirement to develop novel adjuvants for vaccines. Traditional Chinese Medicine (TCM) involves the use of natural products that have been utilized by humans for centuries for good health^5^. TCMs contain many bioactive ingredients with beneficial effects, and thus have attracted much attention in recent years^6^. Although herbal medicines are comprised of several ingredients that target multiple organs, it is difficult to pin-point the bioactive compounds by traditional pharmacological methods. In addition, TCMs are not widely used outside of China and other Asian countries because of the lack of scientific data on their mechanisms of drug action^7^. Thus, additional studies are needed to identify the underlying mechanisms.

Systems pharmacology, an emerging area of pharmacology, combines drug-like prediction, absorption, distribution, multiple drug target prediction and network analysis to analyze drugs, drug targets, pathways and drug effects^8^,^9^. This approach, which can be used in the discovery of single bioactive ingredients, can help to identify the mechanisms of drug action^10^,^11^.

Hepatitis B (HB) is an infectious disease caused by the hepatitis B virus (HBV). There are approximately 350 million individuals worldwide that carry the HBV, and China has a high incidence, with approximately 120 million carriers^12^. Chronic HBV infection can lead to hepatitis, cirrhosis and hepatocellular carcinoma (HCC), and effective treatments are lacking. Because of the high morbidity and mortality, hepatitis b vaccination is essential for reducing the carrier rate and preventing viral infection^13^. The currently used genetically engineered hepatitis b vaccine with aluminum hydroxide as the conventional adjuvant has low efficacy, a long waiting period to immune response and large individual differences in immune effects. Approximately 5–10% of individuals with a normal immune system cannot achieve effective immunity against hepatitis b virus infection^14^,^15^,^16^. Therefore, developing a quick and effective adjuvant to enhance hepatitis b vaccine immunogenicity is of great practical value.

*Ophiocordyceps sinensis* (syn. *Cordyceps sinensis*), placed systematically as *Ophiocordycipitaceae, Hypocreales, Hypocreomycetidae, Sordariomycetes, Ascomycota*, is a TCM agent that has been widely used as a folk tonic for nearly one thousand years^17^. It has an overwhelming list of pharmacological properties^18^,^19^,^20^,^21^. As one of the traditional and medical fungi, *O. sinensis* is currently available in China and South East Asia^22^,^23^. *O. sinensis* contains several bioactive components, and many reports have carefully analyzed its make-up of polysaccharides, sterols, nucleosides and protein^21^,^22^,^24^. Many of these components have been found to possess immunological, anti-tumorigenic, anti-oxidative, anti-inflammatory, anti-fatigue, anti-fungal and anti-hypertensive properties, as well as to protect the kidney, liver and lung^25^,^26^. However, there is no systematic analysis of the drug-target network of *O. sinensis*. Therefore, the aim of the current study was to explain how the immunoregulation function was implemented and find a component that can act as an adjuvant of the hepatitis b vaccine through systems pharmacology analysis. Briefly, as seen in Fig. 1, we first use a network pharmacology approach to determine the active ingredients of *O. sinensis*. A molecular-target network was then developed, followed by enrichment analysis and functional classification. After screening, a single component was selected and validated by *in vivo* and *in vitro* experiments.

## Results

### Candidate component identification

It has been difficult to identify the mechanisms of action of different TCM agents due to their complex biochemical make-up, and specific methods to identify the bioactive compounds have not been available until now^27^. In this study, we used oral bioavailability (OB) screening and drug-likeness (DL) property evaluation or drug half-life (HL) prediction to identify the bioactive compounds^7^,^9^. All the ingredients of *O. sinensis* were obtained from the Traditional Chinese Medicines Systems Pharmacology Database and Analysis Platform^28^ and the CancerHSP Database^29^. Twenty potential compounds with OB ≥30%, DL ≥0.18 or HL ≥4 were selected. Additionally, three compounds in the CancerHSP database with potential anti-cancer activities and OB ≥30% were also selected as candidate compounds for further analysis. Duplicate compounds in the two databases were eliminated, yielding 23 readily-absorbed compounds from a total of 40 compounds. The satisfied compounds are presented in Supplementary Table S1.

### Target prediction and functional analysis

**Figure 2:**
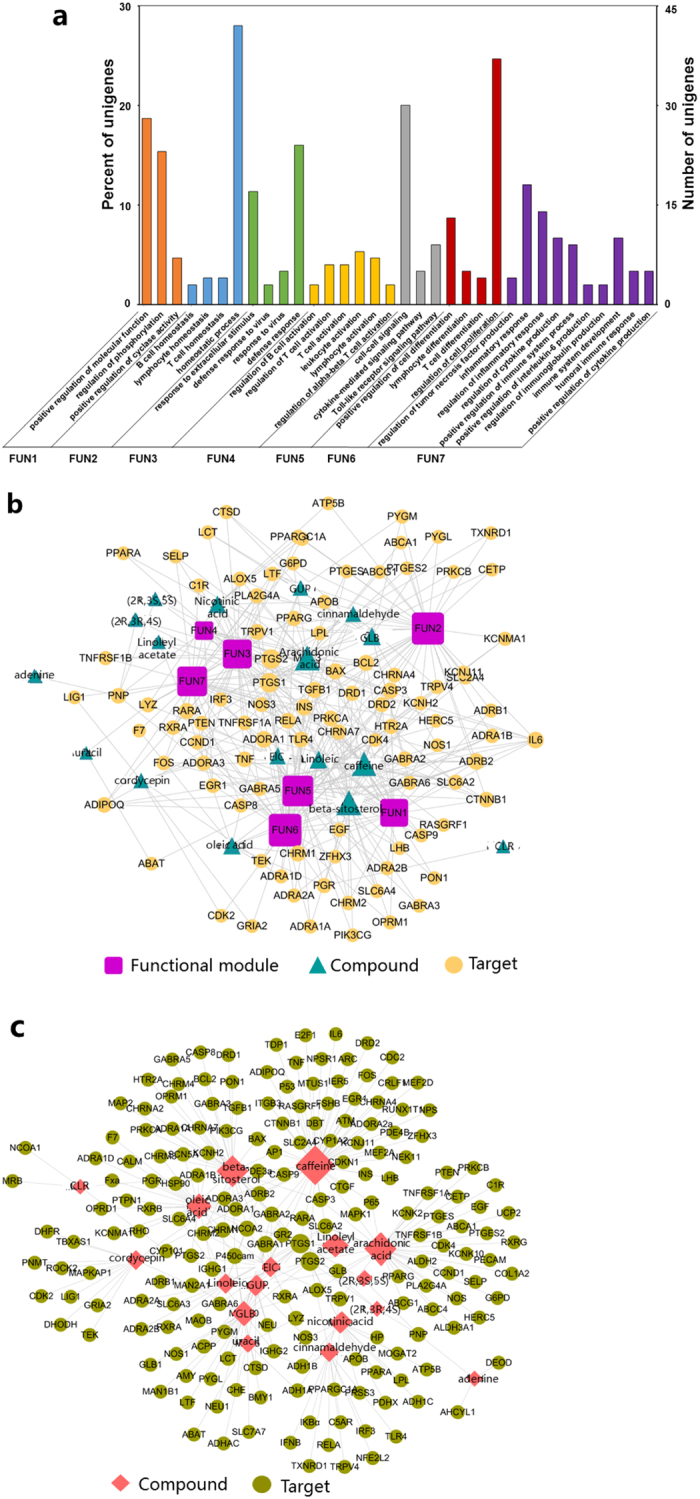
(**a**) Enrichment results of the selected functional mode of *Ophiocordyceps sinensis*. (**b**) Compound-target-function network. A compound is linked to a target if the target protein is hit by the corresponding compound. Similarly, a target is linked to a functional module if the target is involved in the biological process. (**c**) Compound-target relationship. A compound is linked to a target if the protein target is hit by the compound.

Generally, TCMs could prevent diseases through synergistic effects of different compounds and targets. Therefore, the potential therapeutic targets of the multiple compounds were important for its synergistic effects. After eliminating six additional compounds without targets, 190 targets in the database were assembled and ranked for 17 components contained in *O. sinensis*. Detailed enrichment analysis and functional pathway classification showed that the bioactive components were involved in several cellular events, including signal transduction and cell differentiation (Fig. 2a and Supplementary Table S2).

### Network construction

*O. sinensis* exerts extensive biological and pharmacological effects through multiple compound and target interactions. To understand these effects at the systemic level, a compound-target network was constructed based on the candidate components and targets^30^. Figure 2b shows the results of the compound-target-function network, which consisted of 17 compounds, 190 candidate targets and seven functional annotations. The results displayed an average degree of 9.4 per compound and 3.8 per target protein, respectively. With regard to the relationships between compounds and targets, as shown in Fig. 2c, the compound-target network embodied 207 nodes (17 potential compounds and 190 potential targets). The mean degree value (the number of associated targets) of candidate components was 18.1, and 11 components possessed a mean degree value greater than 12, suggesting that most components associated with multiple targets to exert different biological and pharmacological effects. The major components and its target numbers are shown in Table S3. Specifically, components such as caffeine, beta-sitosterol (Sito) and arachidonic acid (AA), which acted on 54, 38 and 38 targets, respectively, were the crucial active components for *O. sinensis* in this network. The second major components are oleic acid, cordycepin and nicotinic acid.

### Immune regulation mode

**Figure 3:**
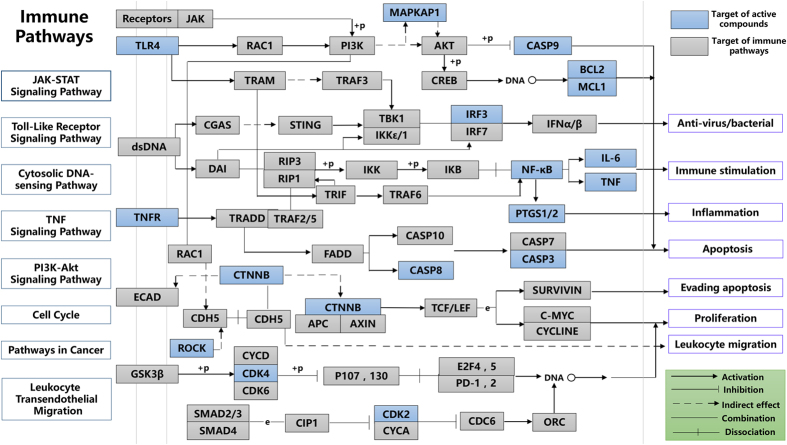
Immune pathways and component targeted modules.

Previous data showed that the targets of *O. sinensis* were involved in immune function that included positive regulation of the immune system to bacterial infection, and T and B cell function. Cytokines also play an important role in the activation and regulation of immune responses. As can be seen from Fig. 3, marked target genes make a contribution to the production of interferon which are detected to connect with anti-virus or bacterial responses. On the other hand, components in *O. sinensis* also possess anti-inflammatory effects during both our prediction and some other studies. For instance, cordycepin was observed to suppress LPS-stimulated release of pro-inflammatory cytokines TNFα and IL-1β through NF-κB pathway^31^; Sito was observed to induce cancer cell line apoptosis through decrease the expression of apoptosis regulator Bcl-2 which makes it a candidate for cancer chemotherapy^32^. Besides, cordycepin was predicted to interact with ROCK which related to leukocyte migration and this may contribute to the formation of germinal center or other cellar immune responses.

There have been many studies in the 3 key components in *O. sinensis*, for example, caffeine was used to defense depression and AA could promote secretion of pro-inflammatory leukotrienes^33^,^34^. After screening the major components of *O. sinensis*, the nucleoside antibiotic cordycepin was selected for further experimental validation based on our adjuvant screen purpose. Cordycepin has received much attention, mostly because of its anti-tumor and antiviral activities in the inhibition of viral DNA/RNA synthesis^35^,^36^,^37^. For example, cordycepin induces apoptosis of tumorigenic Leydig cells in the mouse via several signaling pathways^38^,^39^,^40^. It also plays roles in platelet aggregation and inflammation^36^,^41^,^42^; however, there are few reports on the use of cordycepin as an adjuvant to enhance the immune response. We here in combined cordycepin with HBsAg to generate a new vaccine and tested its immunologic activity in mice.

### Cordycepin improves HBsAg-specific antibody production without side effects

**Figure 4:**
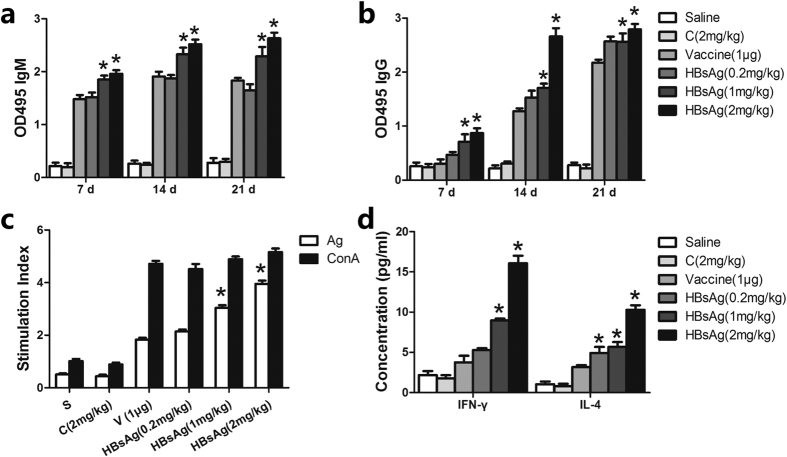
Effects of cordycepin (C) on serum HBV antibodies, lymphocyte proliferation and cytokine levels in spleen cell supernatants. (**a**) Mice (n = 10 in each group) were injected subcutaneously on days 0, 7 and 14 with saline, vaccine (1 μg/mouse) or HBsAg (1 μg/mouse) adjuvanted with cordycepin (0.2, 1 or 2 mg/kg). Serum samples were collected on days 7, 14 and 21 after the first injection and HBsAg-specific (**a**) IgM and (**b**) IgG were analyzed by ELISA. Splenocytes were collected 15 days after the last injection and stimulated with the corresponding antigen and ConA (5 μg/ml). (**c**) Splenocyte proliferation was measured by the CCK8 method. (**d**) ELISA was used to measure cytokine levels in spleen cell supernatants. The values are presented as the mean ± SEM, *represents p < 0.05 compared with vaccine group.

Serum HBV antibodies were measured by ELISA to determine the effects of the adjuvant on the humoral immune response. As shown in Fig. 4, cordycepin (0.2, 1 and 2 mg/kg) adjuvanted with HBsAg led to an increase in the serum antibody level in a dose-dependent manner. The IgM level remained high on day 21 in the 2 mg/kg cordycepin group but not in the other groups (Fig. 4a). On day 14, the IgG titers induced by 2 mg/kg cordycepin adjuvanted with HBsAg were similar to the vaccine group on day 21 after the last injection (Fig. 4b). However, there was no difference in the IgG level in this group on days 14 and 21. Furthermore, there was no significant different in body weight in mice from all groups (Figure S1a). There was also no apparent histological change in the liver and spleen of mice from all groups on day 28 by haematoxylin-eosin staining and routine light microscopy (Figure S1b).

### Cordycepin adjuvanted vaccine promotes lymphocyte proliferation and cytokine production *in vivo* and *in vitro*

The enhancement of cellular immunity is also an important barrier for HBV infection. HBsAg adjuvanted with 1 or 2 mg/kg cordycepin resulted in significantly higher proliferative activity than that in the other group. The cell activity of the 2 mg/kg cordycepin group was similar to that of the positive control (Fig. 4c), indicating that it elicits an effective cellular immune response. The increased cell supernatant cytokine levels (Fig. 4d) suggest that both Th1 and Th2 cell responses were activated by cordycepin.

### Lymphocyte differentiation in the spleen after immunization

**Figure 5:**
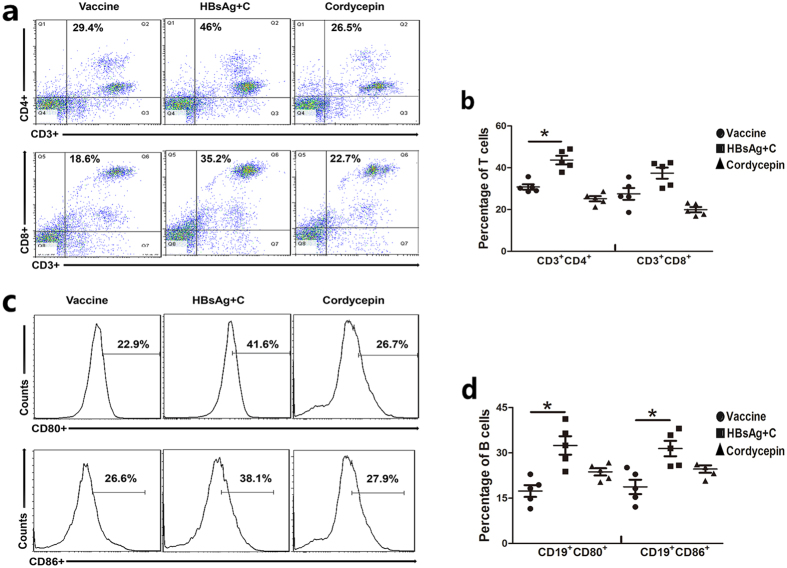
Effects of cordycepin (C) on spleen lymphocyte differentiation. Splenocytes from the cordycepin-adjuvanted (2 mg/kg) group were collected on day 15 after the last injection and analyzed by flow cytometry. (**a**) Percentage of T cells after injection. (**b**) Each point represents a mouse of (**a**). (**c**) Percentage of T cells after injection. (**d**) Each point represents a mouse of (**c**). The numbers in the quadrants represent the percentages. and the line indicates the mean value ± error bars *p < 0.05.

To assess T and B cell differentiation after immunization, spleen cells were collected and analyzed by flow cytometry (Fig. 5). The percentage of CD3^+^CD4^+^Th cells and CD3^+^CD8^+^CTL cells were all increased by the supplementary of cordycepin. The CD80 and CD86 expression on CD19+ B cells were also enhanced in the adjuvant groups, indicating that B cells were activated more effectively. The increase in Th and B cell number indicates that the increase in antibody production and CTL cell number enhanced the cytotoxic effect, which might associate with IFN-γ.

## Discussion

Complex composition of TCMs makes it hard to understand its therapeutic mode from a molecular level. In recent years, several herbal components with different pharmacological activities have been identified as the development of biological analysis; these components constitute a substantial percentage of today’s new drugs^43^,^44^. One of the most commonly used method to investigate new drugs is to start from a disease and search from natural products which could treat it, however, the limitation of technical difficulties and suitable animal models makes it difficult to understand their mode of action^45^,^46^. Recently, systems pharmacology offers a new approach to study bioactive components of TCM agents, as well as their drug-target interactions. Herein, we try to selective adjuvant from *O. sinensis* for its remarkable immunoregulation effects. Although there have been many studies about effective components in *O. sinensis*, the exact action mode was not fully elicited at a system level.

In the present study, with the help of OB, DL and HL screening, 17 active compounds with 190 potential targets were identified in *O. sinensis*. The target enrichment analysis shows that these targets take part in many biological pathways such as cytokine secretion, drug binding, blood circulation and so on. Several targets were also involved in different types of cancer, supporting an earlier study which showed *O. sinensis* extracts to exhibit anti-cancer effects^47^,^48^,^49^. It was not a surprise that several major components have been studied clearly in previous researches. Caffeine is a well-known phosphodiesterase 3 (PDE3) inhibitor and antagonist of adenosine receptors^50^. Caffeine can inhibit the production of tumor necrosis factor alpha (TNF-α) in lipopolysaccharide-stimulated human whole blood^51^ and modulate specific biological parameters associated with depression, thereby preventing the disease^52^,^53^. Interestingly, caffeine can also protect against the production of free radicals^54^. Sito is a functional phytosterol with a chemical structure similar to that of cholesterol^55^. Sito alone or combined with other plant sterols is known to reduce blood cholesterol levels by blocking cholesterol absorption^56^,^57^. Sito has also been reported to possess antimicrobial activity^58^. AA, an unsaturated fatty acid that modulates the activities of various ion channels, functions as a second messenger^59^. As the adjuvant selection purpose, we choose all immune related function to get a component-target-function network. Results show that all components participate in these processes. Then, an immune-related pathway was constructed to further dissect the potential immunoregulation activities of components in *O. sinensis*. Cordycepin was finally selected because of its significant functions to suppress inflammation and regulate leukocyte migration as well as cell proliferation in the networks. Although it was not the only key component in *O. sinensis*, other components may interference with nutrient absorption or inhibit cytokine secretion, may have an adverse effect on healthy people or just suppress immune responses.

There have been many studies on cordycepin these days, mainly concentrate on its anti-tumor and immunoregulation effects^60^,^61^,^62^, but few reports pay attention to its adjuvanticity to vaccines. In the experimental validation part, cordycepin triggered IgM and IgG antibody responses in mice by 7 days in a dose dependent manner after combination with a hepatitis b vaccine. The high cordycepin group showed no difference in IgG titers between days 14 and 21 suggesting that the third injection might be unnecessary. Certainly, this would reduce the overall cost and the immune response time. Moreover, primary safety evaluation demonstrates that a high dose of cordycepin adjuvanted with HBsAg accelerated antibody production more rapidly than the existing vaccine without any side effects.

Enhancement of celluar immunity is also important for defense of HBV infection, especially T cell responses and T cell related cytokines which involved in B cell activation and antibody production^63^. The Th1-type of cellular immune response damages the virus, whereas the main function of the Th2-type of cellular immune response is to promote B cell production of antibodies. IFN-γ is a multifunctional cytokine that is primarily produced by Th1 cells, CTLs and natural killer cells with antiviral and immunomodulatory activities^64^,^65^,^66^. IL-4 controls Th2 differentiation and plays an important role in antibody class switching^67^,^68^. Enhanced cytokine production by cordycepin suggests that it accelerates Th1 and Th2 cell responses at the same time. Jeong *et al*. also demonstrated that cordycepin increases the levels of different cytokines *in vitro*, contributing to the enhancement of immunity^69^. The Th2 cell activation results in B cell maturation which consistent with the B cell classification experiments.

To sum it up, the systems pharmacology analysis offers us a new way to discover effective adjuvants from certain TCMs. Here we get a comprehensive understanding of function mechanism of *Ophiocordyceps sinensis* in a molecular-target level. In the component-target network, the active components showed enormous polypharmacology, and after pathway enrichment analysis, multiple targets were found to participate in many physiological activities, including ion transport and stimulus response. To enhance the immune response, we selected cordycepin as a potential adjuvant of the hepatitis B vaccine. The results of our experiments showed that cordycepin significantly enhances humoral and cellular immunity in BALB/c mice without any side effects. Our results lay the foundation for the investigation of new combined vaccines.

## Methods

### Dataset construction

All the ingredients of *Ophiocordyceps sinensis* were extracted from the Traditional Chinese Medicine Systems Pharmacology Database and Analysis Platform (http://lsp.nwsuaf.edu.cn/tcmsp.php) and the Anticancer Herbs Database of Systems Pharmacology (http://lsp.nwsuaf.edu.cn/CancerHSP.php). Twenty potential compounds with OB ≥30%, DL ≥0.18 or HL ≥4 were selected. Additionally, three compounds in the CancerHSP Database with potential anti-cancer activities and OB ≥30% were also selected as candidate compounds for further analysis. Duplicate compounds in the two databases were eliminated, yielding 23 readily-absorbed compounds from a total of 40 compounds. After eliminating the additional compounds without targets, the 190 targets in the database were assembled and ranked for the 17 components present in *O. sinensis*.

### Network construction

To obtain a better understanding of the complex relationships between the compounds and targets, a network was constructed. The candidate compounds and potential targets were used to construct the compound-target network. The network was generated and analyzed by Cytoscape 3.2.1^70^. The degree of a node defined the number of targets connected to it, which indicated the importance of the node in the network.

### Pathway enrichment analysis for selected targets

The Database for Annotation, Visualization and Integrated Discovery (DAVID, https://david.ncifcrf.gov/home.jsp, version 6.7) was used for GO enrichment analysis.

### Vaccine, adjuvant and antibodies

The hepatitis B vaccine (consisting of HBsAg and aluminium adjuvant) and HBsAg were a generous gift from the Dalian Hissen BioPharm Co., Ltd. (Dalian, China). Cordycepin was purchased from Beijing Century Bioko Bio-technology Co. (Beijing, China). The endotoxin levels of these reagents were <0.1 EU/ml as determined by an endotoxin detection assay (Tachypleus Amebocyte Lysate, Xiamen, China). Fluorescein isothiocyanate (FITC)-conjugated anti-mouse CD3, phycoerythrin (PE)-conjugated anti-mouse CD4, PE/Cy5-conjugated anti-mouse CD8a, FITC-conjugated anti-mouse CD80, FITC-conjugated anti-mouse CD86 and PE-conjugated anti-mouse CD19 antibodies were all purchased from BioLegend (BioLegend, San Diego, CA, USA).

### Mouse immunization and sample collection

Female BALB/c mice at 4–6 weeks of age were obtained from the Shanghai SLAC Laboratory Animal Center and assigned to one of five experimental groups with ten mice in each group. They were maintained under specific-pathogen-free conditions at Fudan University. The principles of laboratory animal care were followed precisely. All procedures were conducted according to the guidelines established by the National Institutes of Health, and every effort was made to minimize discomfort and suffering. This study was approved by the Animal Experiment Committee of Fudan University. Each mouse was injected subcutaneously on days 0, 7 and 14 with normal saline, cordycepin (2 mg/kg), vaccine (1 μg), or HBsAg (1 μg) adjuvanted with cordycepin at 0.2 mg/kg, 1 mg/kg or 2 mg/kg. Serum samples were obtained on days 7, 14 and 21 after the first injection for the measurement of cytokines and HBV-specific antibodies. The body weight of each mouse was measured on days 7, 14, 21 and 28 after the first injection, the liver and spleen were collected on day 28 for routine histology and haematoxylin-eosin staining to evaluate the side effects of the new adjuvant. At 15 days after the last injection, the mice were sacrificed, and splenocytes were isolated to determine the cytokine production and cellular proliferation, as well as the cell differentiation.

### Measurement of serum antibodies

The HBV-specific serum antibody titers were measured by ELISA. Briefly, 96-well plates (Grenier, Frickenhausen, Germany) were coated with 2 μg/ml of HBsAg overnight at 4 °C. After blocking with 5% bovine serum albumin in phosphate-buffered saline containing Tween-20 (PBST), 100 μl/well of serially-diluted serum samples from the immunized animals was added, following by incubation with a horseradish peroxidase (HRP)-conjugated goat anti-mouse IgG or IgM antibody (Santa Cruz Biotechnology, Santa Cruz, CA, USA). After washing, 100 μl/well of TMB substrate (BD Biosciences, San Diego, CA, USA) was added, and the reactions were terminated by the addition of 50 μl/well of 2 M H_2_SO_4_. The absorbance was read at 495 nm on a microplate reader (Molecular Devices, Sunnyvale, CA, USA).

### Lymphocyte proliferation assay

Spleen cells were collected from the immunized mice under aseptic conditions and adjusted to a final concentration of 10^6 ^cells/ml in complete medium (RPMI 1640 supplemented with 0.05 mM β-mercaptoethanol, 100 UI/ml penicillin, 100 g/ml streptomycin and 10% FBS). Approximately 1 × 10^6^ cells from each group were stimulated with the corresponding reagent (100 μl saline for saline group, 500 ng cordycepin for cordycepin group, 100 ng vaccine for vaccine group and 100 ng HBsAg plus 500 ng cordycepin for the other 3 groups) and ConA (5 μg/ml). After 2 days of incubation (under humidified conditions of 5% CO_2_ and 37 °C), 10 μl of CCK-8 solution (Beyotime Biotechnology, China) was added 4 h before the end of the incubation period. The absorbance was measured at 450 nm using a microplate reader (Molecular Devices). The stimulation index = (test OD- blank OD)/(negative OD-blank OD). An SI ≥2 indicated positive results.

### IFN-γ and IL-4 detection

Approximately 1 × 10^6^ spleen cells were stimulated with the corresponding antigen for 24 h, and the supernatant was analyzed by ELISA. IFN-γ and IL-4 were measured using mouse-specific IL-4 and IFN-γ ELISAs (Dakew, Shenzhen, China) according to the manufacturer’s instructions. Briefly, 100 μl/well of the diluted cytokine standard and the sample was added. Thereafter, 50 μl/well of the biotinylated antibody was added and incubated for 90 min at 37 °C. After washing five times, 100 μl/well of diluted streptavidin-HRP was added and incubated for 30 min at 37 °C. The plate was then washed and 100 μl/well of TMB was added. After incubation in the dark for 15 min, 100 μl/well of stop solution was added, and the absorbance was measured at 450 nm.

### FACS analysis

Approximately 1 × 10^6^ spleen cells were collected, resuspended in 100 μl of PBS and incubated with 1.5 μl of PE-conjugated anti-mouse CD4, PE/Cy5-conjugated anti-mouse CD8a and FITC-conjugated anti-mouse CD3, PE-conjugated anti-mouse CD19 and FITC-conjugated anti-mouse CD80, or FITC-conjugated anti-mouse CD86 antibodies (all from BioLegend) in the dark on ice for 30 min. The cells were then washed and analyzed using a FACSCalibur flow cytometer and CellQuest software (BD Biosciences).

### Statistical analysis

Data are expressed as the means ± standard error of mean (mean ± SEM). A two-sided Student’s *t*-test for paired or unpaired data was performed using SPSS19 software (SPSS Inc., Chicago, IL, USA). Differences between experimental and control samples with a *p* < 0.05 were considered statistically significant.

## Additional Information

**How to cite this article:** Wang, J. *et al*. Systems Pharmacology-based strategy to screen new adjuvant for hepatitis B vaccine from Traditional Chinese Medicine *Ophiocordyceps sinensis. Sci. Rep.* **7**, 44788; doi: 10.1038/srep44788 (2017).

**Publisher's note:** Springer Nature remains neutral with regard to jurisdictional claims in published maps and institutional affiliations.

## Supplementary Material

Supplementary Information

## Figures and Tables

**Figure 1:**
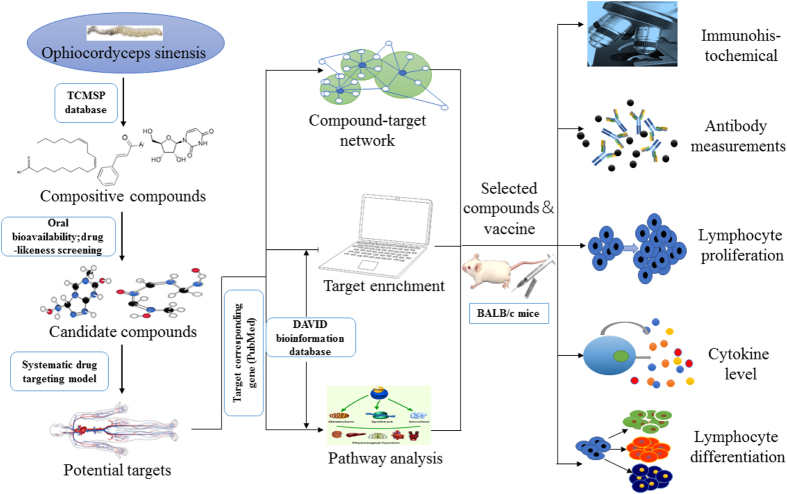
Workflow of the systems pharmacology approach. Pictures of potential targets (human body) and pathway analysis (different body parts and cell components) were prepared with Portable Pathway Builder (version 2.0, http://pathway-builder-tool.software.informer.com). Photos of *Ophiocordyceps sinensis*, BALB/c mice, injector and microscope were token in our lab. All the other pictures were prepared with Microsoft PowerPoint (Version 16.0.6366.2056).
