# Supplementary material for: Systems Pharmacology-based strategy to screen new adjuvant for hepatitis B vaccine from Traditional Chinese Medicine Ophiocordyceps sinensis
Source: Sci Rep. 2017 Mar 20;7:44788. doi: 10.1038/srep44788 (PMC5357901; doi:10.1038/srep44788)
Supplement: Supplementary Information [file srep44788-s1.pdf]

**Title:**

# **Systems Pharmacology-based strategy to screen new adjuvant for hepatitis B vaccine from Traditional Chinese Medicine *Ophiocordyceps sinensis***

Jingbo Wang[1+], Rui Liu[1+], Baoxiu Liu[1], Yan Yang[1], Jun Xie[1]*, Naishuo Zhu[1]*

[1] Laboratory of Molecular Immunology, State Key Laboratory of Genetic Engineering, Institute of Biomedical Science, School of Life Sciences, Fudan University, Shanghai 200438, China.

*Correspondence and requests for materials should be addresses to Naishuo Zhu (Email: nzhu@fudan.edu.cn) and J.X. (Email: xiejun@fudan.edu.cn)

[+] These authors contributed equally to this work.

Table S1. Information for 23 candidate compounds in *Ophiocordyceps sinensis*, including their OB, DL, HL, degree and structures.

| | Molecullar ID | Compuond | OB(%) | DL | HL | Structure |
|---|---|---|---|---|---|---|
| 1 | MOL001308 | oleic acid | 33.13 | 0.14 | 5.39 | 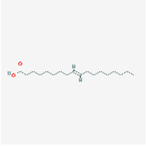 |
| 2 | MOL000131 | EIC | 41.9 | 0.14 | 7.5 | 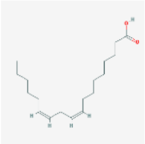 |
| 3 | MOL001439 | arachidonic acid | 45.57 | 0.2 | 4.39 | 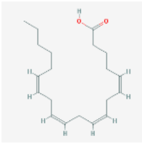 |
| 4 | MOL001645 | Linoleyl acetate | 42.1 | 0.2 | 7.48 | 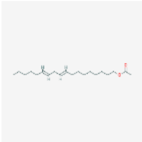 |
| 5 | MOL001744 | uracil | 42.53 | 0.02 | 11.77 | 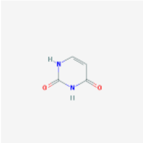 |
| 6 | MOL001788 | adenine | 62.81 | 0.03 | 13.33 | 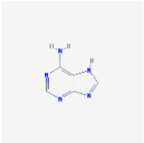 |
| 7 | MOL002225 | Styrone | 38.35 | 0.02 | 4.38 | 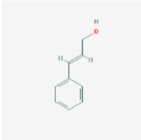 |

| | | | | | | |
|---|---|---|---|---|---|---|
| 8 | MOL000358 | beta-sitosterol | 36.91 | 0.08 | 5.36 | 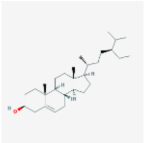 |
| 9 | MOL003973 | caffeine | 89.46 | 0.08 | 13.64 | 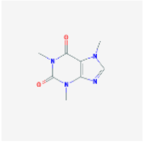 |
| 10 | MOL000421 | nicotinic acid | 47.65 | 0.02 | 11.98 | 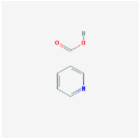 |
| 11 | MOL004668 | GLB | 47.71 | 0.04 | 11 | 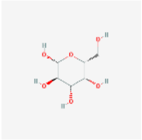 |
| 12 | MOL005367 | GUP | 43.04 | 0.04 | 11.1 | 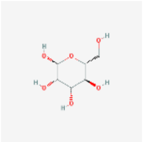 |
| 13 | MOL011169 | Peroxyergosterol | 44.39 | 0.82 | 4.06 | 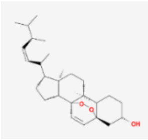 |
| 14 | MOL000749 | Linoleic | 41.9 | 0.14 | 5.27 | 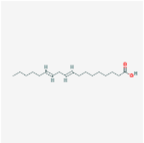 |
| 15 | MOL000857 | NCA | 71.13 | 0.02 | 11.89 | 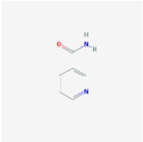 |

| | | | | | | |
|---|---|---|---|---|---|---|
| 16 | MOL008998 | cerevisterol | 39.52 | 0.77 | 5.08 | 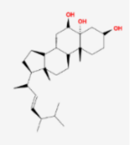 |
| 17 | MOL008999 | cholesteryl palmitate | 31.05 | 0.45 | 7.93 | 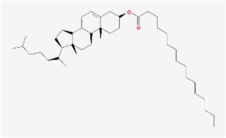 |
| 18 | MOL009000 | (2R,3S,5S)-5-(6-aminopurin-9-yl)-2-(hydroxymethyl)oxolan-3-ol | 30.13 | 0.15 | 6.41 | 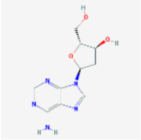 |
| 19 | MOL009001 | (2R,3R,4S)-2-(6-aminopurin-9-yl)-4-(hydroxymethyl)oxolan-3-ol | 38.44 | 0.16 | 6.83 | 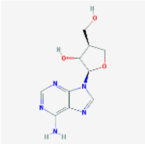 |
| 20 | MOL000953 | CLR | 37.87 | 0.68 | 4.52 | 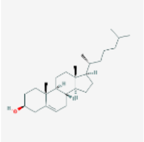 |
| 21 | MOL000991 | cinnamaldehyde | 31.99 | 0.02 | 4.73 | 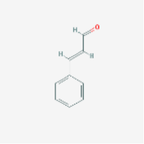 |
| 22 | HB0462 | cordycepin | 36.83 | - | - | 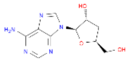 |
| 23 | HBO462 | cordycedi peptide A | 63.12 | - | - | 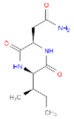 |

Table S2. Candidate target proteins associated biological processes of *Ophiocordyceps sinensis*

| Functional classification | Biological process | Genes | Count | P-Value | FDR |
|---|---|---|---|---|---|
| molecular function | positive regulation of molecular function | OPRM1, DRD1, TNF, ADORA3, ADORA2A, DRD2, TLR4, ADORA1, TGFB1, CASP9, INS, BCL2, ADRA2A, NOS3, CHRNA7, ADRA2B, EGF, IL6, RELA, ADRB2, CCND1, ADRB1, RASGRF1, CHRM2, BAX, CHRM1, PON1, HTR2A | 28 | 3.39E-10 | 5.98E-07 |
| | regulation of phosphorylation | PRKCA, IL6, DRD1, TNF, ADORA2A, HERC5, TLR4, ADORA1, ADIPOQ, PTEN, TGFB1, CASP3, CCND1, ADRB2, INS, BAX, BCL2, CHRM1, ADRA2A, CHRNA7, ADRA2B, EGF, HTR2A | 23 | 1.14E-08 | 2.02E-05 |
| | positive regulation of cyclase activity | OPRM1, ADRB2, DRD1, ADRB1, ADORA3, ADORA2A, NOS3 | 7 | 4.12E-05 | 0.072788 |
| homeostasis | B cell homeostasis | CASP3, BCL2, BAX | 3 | 0.006454 | 10.80384 |
| | lymphocyte homeostasis | CASP3, BCL2, BAX, TGFB1 | 4 | 0.003708 | 6.348592 |
| | T cell homeostasis | CASP3, BCL2, BAX, TGFB1 | 4 | 0.001594 | 2.778044 |
| | homeostatic process | DRD1, PTGES2, ADORA2A, TRPV1, DRD2, ATP5B, PPARG, HP, ABCA1, ADORA1, KCNJ11, TGFB1, CTNNB1, CASP3, APOB, SLC2A4, INS, BCL2, CHRNA4, TRPV4, LTF, CHRNA7, CETP, KCNMA1, PRKCA, LPL, IL6, ADIPOQ, PPARGC1A, ABCG1, PRKCB, PLA2G4A, ADRB2, G6PD, ADRB1, PYGM, PYGL, BAX, ADRA1B, TXNRD1, KCNH2, HTR2A | 42 | 6.21E-18 | 1.10E-14 |
| response to stimulation | response to extracellular stimulus | PPARA, PTGS2, RXRA, RELA, PPARG, PPARGC1A, PTEN, ADIPOQ, TGFB1, FOS, CCND1, PLA2G4A, BCL2, CTSD, RARA, ALOX5, LCT | 17 | 3.37E-09 | 5.95E-06 |
| | defense response to virus | IL6, RELA, BCL2 | 3 | 0.020872 | 31.09495 |
| | response to virus | IL6, TNF, RELA, BCL2, IRF3 | 5 | 0.034413 | 46.11536 |
| | defense response | SELP, IL6, DRD1, TNF, ADORA3, ADORA2A, TRPV1, RXRA, RELA, PPARG, GABRA5, LYZ, C1R, HP, TLR4, ADORA1, TGFB1, FOS, TNFRSF1A, TNFRSF1B, INS, BCL2, LTF, ALOX5 | 24 | 3.63E-07 | 6.41E-04 |

| Functional classification | Biological process | Genes | Count | P-Value | FDR |
|---|---|---|---|---|---|
| cell activation | regulation of B cell activation | CASP3, IL6, TGFB1 | 3 | 0.112049 | 87.73387 |
| | regulation of T cell activation | CASP3, IL6, ADORA2A, RARA, PNP, TGFB1 | 6 | 0.010267 | 16.65819 |
| | T cell activation | EGR1, INS, BCL2, BAX, TGFB1, CTNNB1 | 6 | 0.013819 | 21.78456 |
| | leukocyte activation | EGR1, INS, BCL2, BAX, CHRNA4, TLR4, TGFB1, CTNNB1 | 8 | 0.019523 | 29.39959 |
| | lymphocyte activation | EGR1, INS, BCL2, BAX, CHRNA4, TGFB1, CTNNB1 | 7 | 0.024889 | 35.91965 |
| | regulation of alpha-beta T cell activation | ADORA2A, RARA, PNP | 3 | 0.061627 | 67.47392 |
| cell signalling | cell-cell signaling | DRD1, SLC6A2, ADORA2A, DRD2, SLC6A4, ADORA1, CTNNB1, PGR, INS, TEK, TRPV4, CHRNA4, ADRA2B, PRKCA, KCNMA1, IL6, GABRA2, NOS1, GABRA3, GABRA6, GABRA5, MAPK1, GRIA2, CHRM1, ADRA1B, ABAT, ADRA1A, LHB, ADRA1D, HTR2A | 30 | 2.13E-11 | 3.77E-08 |
| | cytokine-mediated signaling pathway | TNFRSF1A, IL6, TNF, RELA, ADIPOQ | 5 | 0.007912 | 13.08682 |
| | Toll-like receptor signaling pathway | PIK3CG, MAPK1, FOS, IL6, TNF, RELA, CASP8, IRF3, TLR4 | 9 | 0.002337 | 2.709692 |
| cell proliferation and differentiation | positive regulation of cell differentiation | LPL, IL6, APOB, INS, DRD2, RELA, BCL2, PPARG, RARA, ZFHX3, PNP, TGFB1, CTNNB1 | 13 | 1.05E-05 | 0.018505 |
| | lymphocyte differentiation | EGR1, BCL2, BAX, TGFB1, CTNNB1 | 5 | 0.028776 | 40.28325 |
| | T cell differentiation | EGR1, BCL2, TGFB1, CTNNB1 | 4 | 0.036622 | 48.25183 |
| | regulation of cell proliferation | OPRM1, TNF, PTGS2, ADORA2A, DRD2, PTGS1, PPARG, ADORA1, PTEN, PNP, TGFB1, CTNNB1, PGR, CASP3, PTGES, INS, BCL2, TEK, ADRA2A, NOS3, CHRNA7, EGF, PRKCA, IL6, RELA, RXRA, CDK4, CDK2, MAPK1, ADRB2, CCND1, PLA2G4A, CHRM1, BAX, ADRA1A, ADRA1D, HTR2A | 37 | 2.54E-13 | 4.49E-10 |

| Functional classification | Biological process | Genes | Count | P-Value | FDR |
|---|---|---|---|---|---|
| immune response | regulation of tumor necrosis factor production | RARA, CHRNA7, TLR4, ADIPOQ | 4 | 0.004964 | 8.41193 |
| | inflammatory response | SELP, IL6, TNF, ADORA3, ADORA2A, TRPV1, RXRA, RELA, LYZ, C1R, TLR4, ADORA1, TGFB1, FOS, TNFRSF1A, TNFRSF1B, INS, ALOX5 | 18 | 1.42E-07 | 2.50E-04 |
| | regulation of inflammatory response | PRKCA, IL6, PTGS2, ADORA2A, PPARG, TLR4, ADORA1, ADIPOQ, TNFRSF1A, TNFRSF1B, PLA2G4A, ADRB2, INS, CHRNA7 | 14 | 2.25E-12 | 3.97E-09 |
| | regulation of cytokine production | IL6, TNF, INS, RELA, PPARG, RARA, CHRNA7, TLR4, ADIPOQ, TGFB1 | 10 | 2.05E-04 | 0.361505 |
| | positive regulation of immune system process | MAPK1, IL6, RELA, RARA, C1R, TLR4, F7, PNP, TGFB1 | 9 | 0.005464 | 9.220997 |
| | positive regulation of interleukin-6 production | IL6, TNF, TLR4 | 3 | 0.018923 | 28.63201 |
| | regulation of immunoglobulin production | IL6, TNF, TGFB1 | 3 | 0.034176 | 45.88132 |
| | immune system development | EGR1, G6PD, TNF, BCL2, BAX, LIG1, PPARG, CASP8, TGFB1, CTNNB1 | 10 | 0.003988 | 6.812011 |
| | humoral immune response | IL6, TNF, BCL2, LTF, C1R | 5 | 0.012001 | 19.1997 |
| | positive regulation of cytokine production | IL6, TNF, RARA, TLR4, TGFB1 | 5 | 0.018577 | 28.18662 |

Table S3. Major components and related targets

| Components | Target number | Genes |
|---|---|---|
| Caffeine | 54 | ADORA1, PTGS1, PTGS2, PDE3a, PDE4B, GABRA1, ADORA2a, GR2, ADRB2, P65, FOS, CDKN1, BAX, CASP9, EGR1, MAPK1, TNF, AP1, IL6, CASP3, P53, TDP1, RUNX1T1, CDC2, CYP1A2, CTNNB1, ITGB3, SLC2A4, E2F1, ATM, INS, KCNJ11, ADORA2A, RASGRF1, CHRNA4, ADIPOQ, ARC, SLC6A2, CTGF, GABRA2, FSHB, DBT, LHB, NPSR1, NPS, ADORA3, ZFHX3, CRLF1, DRD2, MTUS1, NEK11, MEF2A, MEF2D, IER5 |
| Beta-sitosterol | 38 | PGR, NCOA2, PTGS1, PTGS2, HSP90, PIK3CG, KCNH2, PRKCA, DRD1, CHRM3, CHRM1, SCN5A, GABRA2, CHRM4, PDE3a, HTR2A, GABRA5, ADRA1A, GABRA3, CHRM2, ADRA1B, ADRB2, CHRNA2, SLC6A4, OPRM1, GABRA1, CHRNA7, P450cam, BCL2, BAX, CASP9, AP1, CASP3, CASP8, PRKCA, TGFB1, PON1, MAP2 |
| Arachidonic acid | 38 | PTGS1, PTGS2, RARA, TRPV1, RXRG, SLC6A2, PTEN, SELP, PTGES, GLB, ALDH2, ABCA1, ALDH3A1, UCP2, C1R, CETP, ABCG1, ABCC4, KCNK10, TNFRSF1B, PTGES2, P65, CCND1, MAPK1, EGF, CDK4, CASP3, HERC5, PPARG, G6PD, TNFRSF1A, PRKCB, PECAM, NOS, ALOX5, PLA2G4A, KCNK2, COL1A2 |
| GLB | 24 | PTGS1, NOS1, ALOX5, PTGS2, GABRA2, GABRA1, PYGM, CTSD, LTF, CHE, AMY, LCT, P450cam, GLB1, IGHG1, IGHG2, NEU1, BMY1, PYGL, ACPP, GR2, GABRA6, MAN1B1, MAN2A1 |
| oleic acid | 24 | PTGS1, PTGS2, RARA, NCOA2, IGHG1, RHO, CHRM3, KCNH2, CHRM1, SCN5A, Fxa, F7, OPRD1, PDE3a, ADRA1B, PTPN1, ADRB2, ADRA1D, HSP90, RXRB, KCNMA1, CALM, PGR, P450cam |
| cordycepin | 17 | TEK, TBXAS1, TBXAS1, DHODH, MAN2A1, ADORA1, GRIA2, DHFR, LIG1, PNMT, DHFR, ROCK2, CDK2, DHFR, DHFR, MAPKAP1, ADORA3 |
| nicotinic acid | 16 | PTGS1, PTGS2, ADH1B, ADH1C, ADH1A, LYZ, PRSS3, APOB, LPL, NOS3, PPARA, ATP5B, HP, PDHX, MOGAT2, PPARGC1A |
| GUP | 15 | PTGS1, NOS3, PTGS2, GABRA1, PYGM, CTSD, LCT, GLB, IGHG1, IGHG2, NEU, GR2, MAN2A1, CYP101, ACPP |

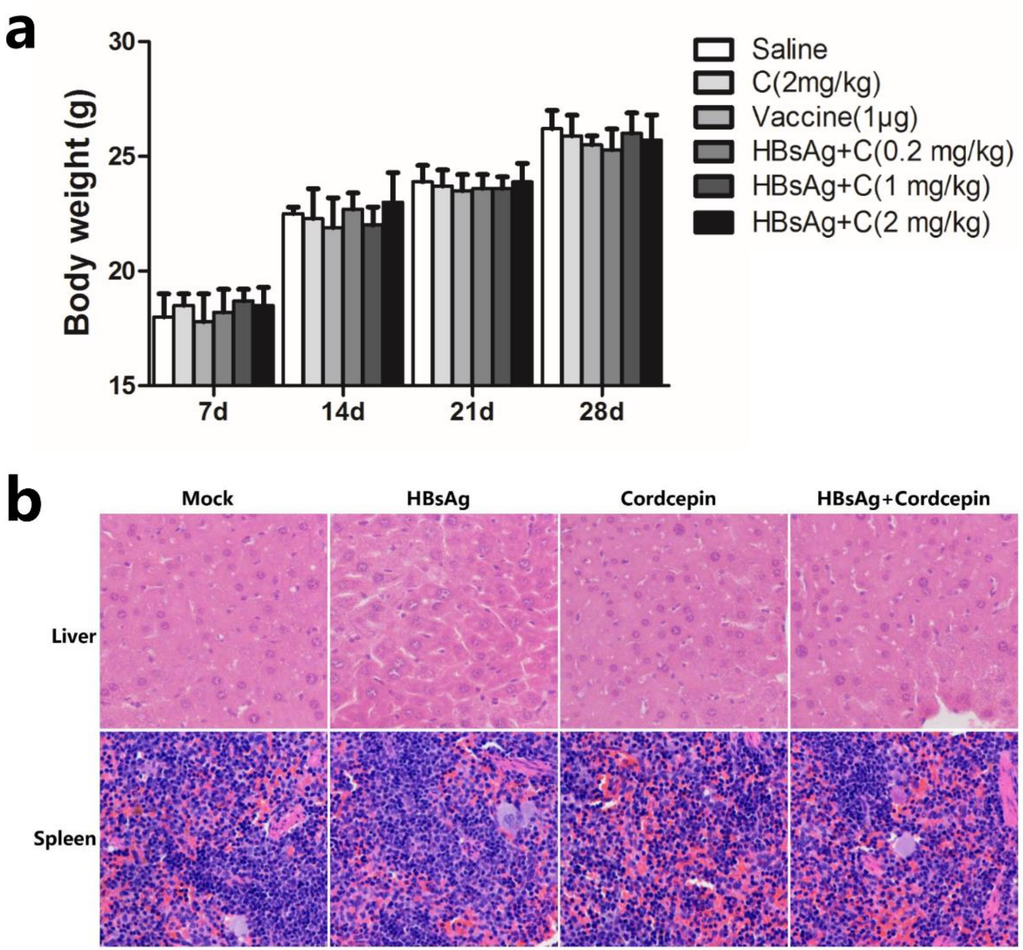


Figure S1. Preliminary safety evaluation of cordycepin. Mice (n = 10 in each group) were injected subcutaneously on days 0, 7 and 14 with saline, vaccine (1 μg/mouse) or HBsAg (1 μg/mouse) adjuvanted with cordycepin (0.2, 1 or 2 mg/kg). (a) Body weight was determined at the indicated time points. (b) Representative photomicrographs of the liver and spleen are shown at a magnification of × 200 (haematoxylin-eosin staining). The values are presented as the mean ± SEM,
